# Evaluation of steroidogenic capacity after follicle stimulating hormone stimulation in bovine granulosa cells of Revalor 200^®^ implanted heifers

**DOI:** 10.1186/2049-1891-5-2

**Published:** 2014-01-07

**Authors:** Andrea D Stapp, Craig A Gifford, Dennis M Hallford, Jennifer A Hernandez Gifford

**Affiliations:** 1 Department of Animal Science, Oklahoma State University, Stillwater, OK 74078, USA; 2 Department of Animal and Range Science, New Mexico State University, Las Cruces, NM 88003, USA; 3 Department of Animal Science, 114B Animal Science Building, Oklahoma State University, Stillwater, OK 74078, USA

**Keywords:** Bovine, Follicle stimulating hormone, Granulosa cells, Implant, Steroidogenesis

## Abstract

**Background:**

Heifers not used as breeding stock are often implanted with steroids to increase growth efficiency thereby altering hormone profiles and potentially changing the environment in which ovarian follicles develop. Because bovine granulosa cell culture is a commonly used technique and often bovine ovaries are collected from abattoirs with no record of implant status, the objective of this study was to determine if the presence of an implant during bovine granulosa cell development impacts follicle stimulating hormone-regulated steroidogenic enzyme expression. Paired ovaries were collected from 16 feedlot heifers subjected to 1 of 3 treatments: non-implanted (n = 5), Revalor 200 for 28 d (n = 5), or Revalor 200 for 84 d (n = 6). Small follicle (1 to 5 mm) granulosa cells were isolated from each pair and incubated with phosphate buffered saline (n = 16) or 100 ng/mL follicle stimulating hormone (n = 16) for 24 h.

**Results:**

Granulosa cells of implanted heifers treated with follicle stimulating hormone produced medium concentrations of progesterone similar (*P* = 0.22) to non-implanted heifers, while medium estradiol concentrations were increased (*P* < 0.10) at 28 and 84 d compared to non-implanted heifers indicating efficacy of treatment. Additionally, real-time PCR analysis in response to follicle stimulating hormone treatment demonstrated a decrease in steroidogenic acute regulatory protein (*P* = 0.05) mRNA expression in heifers implanted for 84 d and an increase in P450 side chain cleavage mRNA in granulosa cells of heifers implanted for 28 (*P* < 0.10) or 84 d (*P* < 0.05) compared to non-implanted females. However, no difference in expression of 3-beta-hydroxysteroid dehydrogenase (*P* = 0.57) and aromatase (*P* = 0.23) were demonstrated in implanted or non-implanted heifers.

**Conclusions:**

These results indicate follicles which develop in the presence of high concentrations of androgenic and estrogenic steroids via an implant tend to demonstrate an altered capacity to respond to follicle stimulating hormone stimulation. Thus, efforts should be made to avoid the use of implanted heifers to study steroidogenesis in small follicle granulosa cell culture systems.

## Background

Combination trenbolone acetate (TBA) and estradiol-17β (E_2_) implants are commonly used in feedlot cattle to increase feed efficiency and muscle mass [1]. However, exposure to exogenous hormones also influences other physiological functions. SJ Jones, RD Johnson, CR Calkins and ME Dikeman [2] demonstrated that implanted bulls had reduced cortisol and testosterone serum concentrations and smaller testicular size compared to non-implanted bulls. These data indicate that combination implants alter adrenal and gonadal steroid production and normal gonadal development.

In females, elevated concentrations of hormones, including estradiol, can alter ovarian function and steroid hormone synthesis. Anabolic agents used to enhance growth inhibit pituitary release of gonadotropins [3] as a result of the androgenic activity as exhibited by TBA [4] or the estrogenic activity [5]. Consequently, implanting developing heifers has marked impacts on reproductive function. Heifers receiving a TBA and E_2_ implant at 84 d of age had delayed puberty and retardation in reproductive tract development [6]. Ewes prenatally treated with testosterone during mid-gestation did not display a delay in onset of puberty but demonstrated absent or disrupted progestogenic cycles, and had larger follicles with prolonged presence [7]. Heifers receiving a TBA implant at estrus or d 13 of the estrous cycle were anestrus for a period of time during growth promotant release, thereafter d 13 implanted heifers remained anestrus due to follicle or luteal cysts [8].

Though steroid implants are not intended for use in breeding females, bovine ovaries are often harvested from abattoirs for GC culture to investigate mechanisms regulating follicle maturation and differentiation. To our knowledge there is no study demonstrating the impact of elevated levels of androgens and estrogens on the developing follicle. Therefore, the objective of this study was to determine if the presence of anabolic and estrogenic steroids impacts follicle stimulating hormone (FSH)-regulated steroidogenic enzyme expression.

## Methods

### Animals

All procedures involving animals were approved by the Oklahoma State University Institutional Animal Care and Use Committee (AG-12-4). Sixteen predominantly Angus heifers (361 kg) were randomly assigned to one of three implant groups: non-implanted (n = 5), implanted for 28 d with a combination implant (200 mg TBA + 20 mg E_2;_ Revalor 200^®^; Intervet, Inc., Millsboro, DE, USA; 28 d; n = 5), and 84 d with Revalor 200^®^ (84 d; n = 6). Assigned heifers were implanted on d 0 (group implanted for 84 d) or d 56 (group implanted for 28 d) and were not re-implanted. Heifers were harvested on d 84 and 85 and paired ovaries were harvested (Robert M. Kerr Food and Agriculture Products Center, Oklahoma State University, Stillwater, OK) from each heifer for GC collection and culture.

### Granulosa cell culture

Small follicle (1 to 5 mm) GC were isolated from ovaries of each animal and each animal’s GC were cultured separately using methods previously described [9]. Follicle size selection was based on the intent of investigating FSH signaling cascades in implanted and non-implanted heifers. Previous observations indicate that 1) recruitment of bovine follicles able to respond to FSH occurs at a diameter of 1 to 3 mm [10]; 2) GC acquire FSH receptors prior to follicular recruitment [11,12]; and 3) GC of recruited follicles express steroidogenic enzyme mRNAs before LH receptor mRNA is detected [13]. Briefly, GC were resupended and washed twice in short-term media (1:1 mixture of Dulbecco’s Modified Eagle Medium (DMEM) and Ham’s F12 containing 0.12 mmol/L gentamycin and 38.5 mmol/L sodium bicarbonate) obtained from Sigma-Aldrich (St. Louis, MO, USA). After the final wash, cells were re-suspended in 0.5 to 2 mL of resuspension medium (serum-free medium with 2.5 mg/mL collagenase and 1 mg/mL DNase) (Sigma-Aldrich) to prevent cell clumping prior to plating. Cell number and viability were determined via hemocytometer using trypan blue dye exclusion. Granulosa cells from each animal were seeded in two-60-mm culture dishes at a density of 5.2 × 10^5^ cells in DMEM complete medium (1:1 DMEM and Ham’s F-12 containing 10% fetal bovine serum, 0.12 mmol/L gentamycin, 2.0 mmol/L glutamine, and 38.5 mmol/L sodium bicarbonate). Incubation of cells occurred at 38.5˚C and 5% CO_2_ and medium was changed every 24 h until cell confluency reached 70-75%. Once confluency was reached, medium and unattached cells were removed. To test how each animal’s GC responded to FSH treatment, one culture dish of GC from each animal were incubated with phosphate buffered saline (PBS; Con; n = 16) in serum free media supplemented with 10^-7^ mol/L testosterone propionate (Sigma-Aldrich) for 24 h, allowing each animal to serve as its own control. The second culture dish was treated with 100 ng/mL purified human FSH (S1AFP-B-3; National Hormone and Peptide Program, National Institute of Diabetes and Digestive and Kidney Diseases, National Institutes of Health, Bethesda, MD, USA; n = 16) in serum free media supplemented with 10^-7^ mol/L testosterone propionate (Sigma-Aldrich) for 24 h. Treatment medium was collected and frozen at -80°C until analysis. Treatments were terminated by removing medium and rinsing cells once with ice cold PBS. Cells were scraped into 1 mL TRIzol (Invitrogen, Grand Island, NY, USA) reagent and stored at -80°C until isolation of RNA.

### RNA extraction and quantitative real-time PCR

RNA was isolated from cultured GC using TRIzol reagent according to the manufacturer’s protocol and stored at -80°C. Integrity of RNA was assessed by visualization of 18S and 28S ribosomal RNA resolved by agarose gel electrophoresis. RNA purity and quantity was determined using a NanoDrop, ND 1000 Spectophometer (Thermo Fisher Scientific, Wilmignton, DE, USA). Purity was determined by 260/280 nm absorbance ratios, absorbance ratios above 1.8 were considered acceptable. Total RNA (1 μg) was treated with 1 μL DNase I Amplification Grade (Invitrogen) to remove genomic DNA contamination following manufacturer’s instructions. First strand cDNA was synthesized from total RNA using oligo(dT) primers and 1 μL Superscript II Reverse Transcriptase (Invitrogen) for each FSH-treated and non-treated samples. Samples were stored at -20°C until analysis. All gene specific primers were designed using Primer3 [14] and synthesized by Integrated DNA Technologies (Coralville, IA, USA). Forward and reverse primer sequences are listed in Table 1. Primers were validated at a concentration of 300 nmol/L using a 7-log dilution curve as previously reported [15].

**Table 1:** Primer sequences used in real-time PCR

		**Sequences of primers (5′-3′)**	
**Gene**	**Accession no.**	**Forward**	**Reverse**
^*1*^*3β-HSD*	NM_174343	CCACACCAAAGCTACGATGA	GCAAGCCAGTACTGCAGAGA
^*2*^*CYP11A1*	NM_176644	AAGTTTGACCCAACCAGGTG	GTGGATGAGGAAGAGGGTCA
^*3*^*CYP19A1*	NM_174305	CAACAGCAGAGAAGCTGGAAGACA	CACCCACAACAGTCTGGATTTCCCT
^*4*^*GAPDH*	NM_001034034	GGGTCATCATCTCTGCACCT	GGTCATAAGTCCCTCCACGA
^*5*^*MRPL19*	NM_001046068	GGAAAGCAGGTTCTTGAGTCC	TGGCATATGGGTCAGCAGTA
^*6*^*PPIA*	XM_002690515	GGTACTGGTGGCAAGTCCAT	GCCATCCAACCACTCAGTCT
^*7*^*STAR*	BC110213	CCCATGGAGAGGCTTTATGA	CGTGAGTGATGACCGTGTCT

^*1*^*3β-HSD* = 3β-hydroxysteroid dehydrogenase.

^*2*^*CYP11A1* = P450 side chain cleavage.

^*3*^*CYP19A1* = aromatase.

^*4*^*GAPDH* = glyceraldehyde-3-phosphate dehydrogenase.

^*5*^*MRPL19* = mitochondrial ribosomal protein L19.

^*6*^*PPIA* = cyclophilin A.

^*7*^*STAR* = steroidogenic acute regulatory protein.

A working solution of cDNA was prepared by diluting 1:10 with DEPC-treated water. Five microliters of cDNA working solution was added to 20 μL master mix containing 13 μL SYBR green and fluorescein mix (Bioline, Taunton, MA, USA) and 0.75 μL of each forward primer (10 μmol/L) and reverse primer (10 μmol/L). Real-time PCR analysis for each sample was carried out in duplicate using a CFX real-time PCR detection system (Bio-Rad Laboratories, Hercules, CA, USA). Standard thermocycler conditions were as follows: 95°C for 10 min, followed by 40 cycles of 95°C for 15 s, 60°C for 30 s, and 72°C for 30 s. Relative fold change in target mRNAs was quantified using the ∆∆Cq method where the FSH ∆∆Cq for each animal was determined by subtracting each animals Con ∆Cq from their FSH ∆Cq [16]. All reverse-transcribed cDNA samples were assayed in duplicate for each gene, and melt curve analyses were performed to ensure specificity of amplification. Melt curve analysis was carried out for 81 cycles with 0.5°C temperature increase from 55°C to 95°C.

To determine the appropriate reference gene to normalize cDNA variability between samples, a panel of three reference genes was analyzed including, glyceraldehyde-3-phosphate dehydrogenase (*GAPDH*), cyclophilin A (*PPIA*), and mitochondrial ribosomal protein L19 (*MRPL19*). The raw Cq values were obtained for each gene in all samples and analyzed using GeNorm (Biogazell qbasePLUS2, Zwijnaarde, Belgium) to determine the most stable normalization factor. The most stable housekeeping gene for target gene normalization was determined to be *GAPDH* and was used as the reference gene [17].

### Radioimmunoassay

Granulosa cell culture medium was analyzed for E_2_ and progesterone (P_4_) by solid-phase radioimmunoassay using components of Siemens Medical Diagnostics Corp (Los Angeles, CA, USA) commercial kits as previously described [9]. The E_2_ concentration in samples of cell culture medium was determined in 200 μL of medium and the specific binding was 62.5%. Detection limit (95% of maximum binding) of the assay was 2 pg/mL. Intra-assay CV for E_2_ was 6.5% for cell culture medium. The P_4_ concentration in samples of GC medium was assayed at 10 μL. The specific binding was 58.8%. Detection limit (95% of maximum binding) of the assay was 0.1 ng/mL. Intra-assay CV for P_4_ was 4.1% for cell culture medium.

### Statistical analysis

Experiments were analyzed by analysis of variance for a completely randomized design in which three treatments were included; non-implanted (n = 5), Revalor 200^®^ for 28 d (n = 5), and Revalor 200^®^ for 84 d (n = 6). Relative fold changes in gene expression for steroidogenic acute regulatory protein (*STAR)*, 3β-hydroxysteroid dehydrogenase (*3β-HSD)*, P450 side chain cleavage (*CYP11A1)*, and aromatase (*CYP19A1)* mRNA, and medium hormonal concentration of P_4_ and E_2_ are presented as the least square means ± standard error of the mean. For all culture experiments, GC from each animal were kept separate and each animal’s GC were subjected to either control treatment or FSH treatment. Thus, fold change values are each animal’s FSH response relative to that animal’s non-treated controls. A value in *CYP11A1* mRNA expression of a non-implanted heifer at least three standard deviations from the mean and a missing fold change for *CYP19A1* in the 84 d treatment group were excluded from statistical analysis. Quantitative real-time PCR data and hormone concentrations were analyzed using the GLM procedures of SAS (SAS Institute, Cary, NC, USA). Data were tested for homogeneity of variance using Hartley’s F max test and *STAR* and E_2_ were corrected by log transformation (log + 3 and log + 1, respectively). When a significant treatment effect was observed, means were separated using the least significant test computed by the predicted difference option of SAS. Statistical significance was set at *P* < 0.10.

## Results and discussion

The anabolic effects of implants are likely a consequence of altering the endogenous hormonal milieu. This concept is supported by the demonstrated increase in plasma GH concentrations in response to E_2_[18] or TBA and E_2_[19] implants. High levels of anabolic hormones can also modulate reproduction as demonstrated by TBA induced anestrus in cows [8,20] and delayed puberty and decreased fertility in TBA plus E_2_ implanted heifers compared to non-implanted controls [6,21].

**Figure 1:**
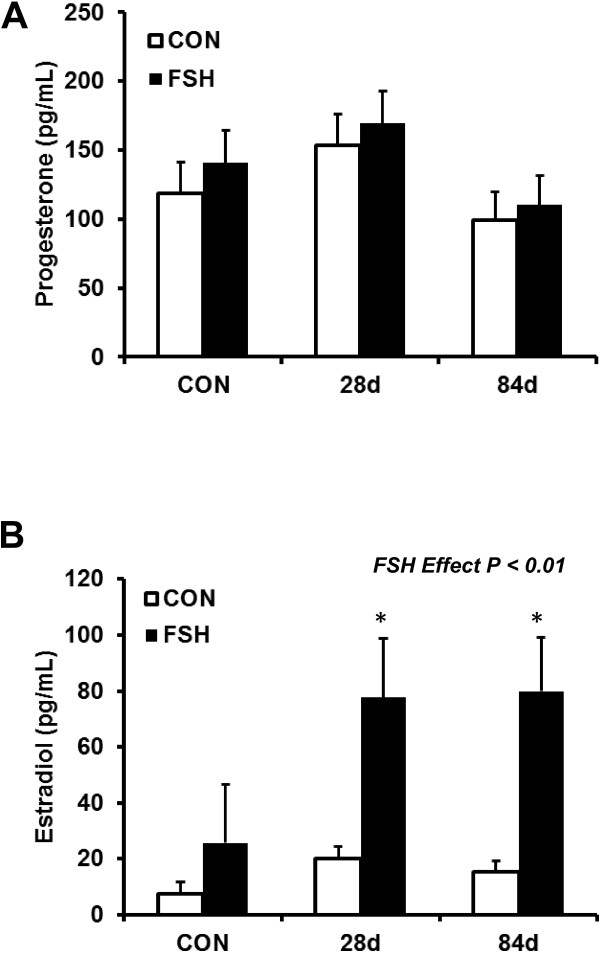
**Anabolic androgens and estrogens did not affect progesterone (P**_**4**_**) production but increased estradiol (E**_**2**_**) concentrations.** Small follicle granulosa cells from non-implanted heifers (n = 5) or heifers implanted for 28 d (n = 5) or 84 d (n = 6) were treated for 24 h with FSH (100 ng/mL, 24 h) or PBS, subsequently cell culture medium were analyzed by RIA. **(A)** Combination implants of TBA and E_2_ did not affect the ability of granulosa cells to produce P_4_. **(B)** Medium E_2_ concentrations were greater in cells exposed to FSH (*P* < 0.01) compared to non-treated controls indicating successful FSH treatment. Additionally, medium E_2_ concentrations increased (*P* < 0.10) in granulosa cells from implanted heifers compared with non-implanted. Concentration is presented as the least square mean ± pooled standard error. Results are compared within FSH treatment group. *P < 0.10 indicates a significant difference when compared with non-implanted heifers.

**Figure 2:**
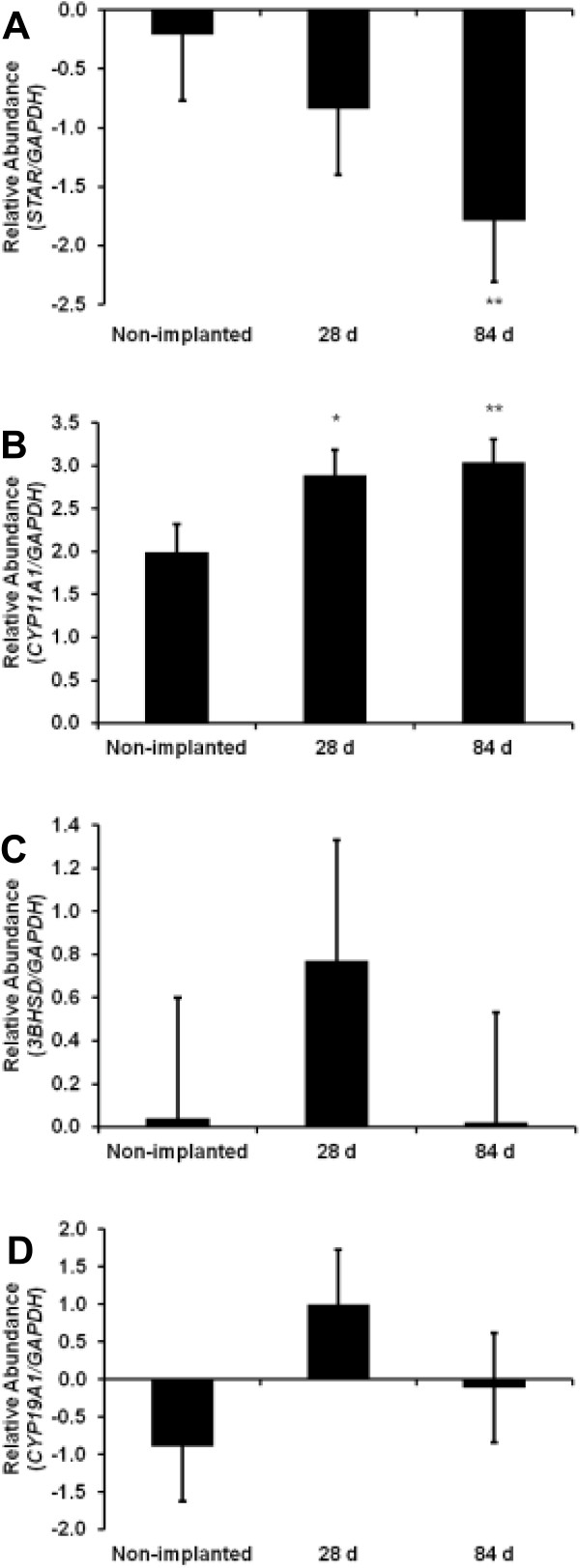
**Expression of key steroidogenic enzymes is altered in FSH stimulated granulosa cells from implanted heifers.** Bovine small follicle granulosa cells were treated as described in Figure 1. Gene expression data are represented as fold change for each group compared to its respective control to demonstrate individual group response to FSH treatment. Fold change of **(A)***STAR*, **(B)***CYP11A1*, **(C)***3βHSD*, and **(D)***CYP19A1* mRNA expression was analyzed by real time-PCR. Statistical significance is presented as the least square mean ± pooled standard error. *P < 0.10 and **P < 0.05 indicates a significant difference when compared with non-implanted heifers.

To determine the steroidogenic capacity of small follicle GC from an environment of elevated anabolic steroids, we first evaluated steroid accumulation in cell culture medium. Hormonal output was analyzed via radioimmunoassay. The ability of cultured GC to synthesize P_4_ was not affected by heifer implant status (*P* = 0.22; Figure 1A). These results are similar to previous studies in which bovine GC were cultured with 200 ng/mL FSH and also did not show an effect on production of P_4_ in cell culture medium compared to controls [22]. As expected, FSH treated GC had increased (*P* < 0.01) concentrations of E_2_ compared to PBS-treated controls (Figure 1B) demonstrating successful induction of the FSH signaling pathway. Additionally, GC from heifers implanted for 28 d or 84 d produced greater concentrations of E_2_ (78 and 80 ± 21 pg/mL, respectively) compared to non-implanted heifers (26 ± 21 pg/mL; *P* < 0.10; Figure 2B) in response to FSH.

Based on the apparent change in estrogen production as a result of implant status and that estrogen production by the follicle is determined by FSH regulation of genes encoding key steroidogenic enzymes, we next evaluated gene expression of the steroidogenic enzymes of non-implanted and implanted heifers in response to FSH. Analysis of steroidogenic enzyme mRNAs of pubertal heifers implanted with TBA and E_2_ in the presence or absence of FSH demonstrated differences in expression as compared to non-implanted heifers. The first rate limiting step in steroid synthesis is the delivery of cholesterol to the inner mitochondrial membrane which is mediated by steroid acute regulatory protein (*STAR*). Expression of *STAR* was reduced (*P* < 0.05) in response to FSH in cells from heifers exposed to TBA and E_2_ for 84 d when compared to non-implanted heifers and heifers implanted for 28 d (Figure 2A). *STAR* is fundamental to the biosynthesis of steroid hormones as it provides cholesterol to the cytochrome P450 side-chain cleavage enzyme (*CYP11A1*). Mitochondrial *CYP11A1* catalyzes the cleavage of the cholesterol side chain to form pregnenolone and this reaction represents the first committed step in steroidogenesis. Follicle stimulating hormone increased mRNA expression of *CYP11A1*, in GC from 28 d (*P* < 0.10) and 84 d (*P* < 0.05) implanted heifers as compared to non-implanted (Figure 2B). Studies indicate that both delivery of cholesterol to the enzyme system and the expression of *CYP11A1* are important factors controlling the rate of steroid hormone synthesis [23] and may contribute to the increase in medium estrogen detected on d 28 and 84. In the female, ovarian androgens and estrogens regulate release of LH and FSH by feedback mechanisms on the hypothalamus and pituitary and it is not unexpected that exposure to anabolic steroids may disrupt this delicate balance. Elevated concentrations of anabolic and estrogenic steroids from the implants did not have a marked effect on mRNA expression of *3β-HSD* (*P* = 0.57; Figure 2C) the enzyme responsible for converting pregnenolone to progesterone. Next, we evaluated *CYP19A1*, the enzyme in granulosa cells responsible for converting androgens to estrogen. However, no change in gene expression was detected for *CYP19A1* in control versus heifers implanted for 28 or 84 d (*P* = 0.22; Figure 1D). This may be explained in part by the relatively short 3 h half-life of *CYP19A1* in FSH-stimulated bovine granulosa cells compared to the more stable 14 h half-life demonstrated for *CYP11A1*[24].

Additionally, although implant status did not result in significant changes in *CYP19A1* mRNA expression, E_2_ concentrations however, were elevated in both FSH treated implanted groups. This is consistent with previous work in cattle showing that minimal regulation in *CYP19A1* gene expression can contribute to measurable differences in E_2_ synthesis [9,25]. Normal follicular development relies on increasing concentrations of E_2_ corresponding with follicle maturation and resultant proliferation and differentiation of GC [26]. Additionally, elevated levels of E_2_ in culture medium improves growth of oocytes from early antral follicles [27] supporting an important role of E_2_ in folliculogenesis. However, elevated exogenous concentration of E_2_ can increase the chance of developing cystic follicles and decreases fertility in heifers [6].

In conclusion, these results indicate that follicles which develop in the presence of high concentrations of androgenic and estrogenic steroids via an implant have an altered ability to respond to FSH stimulation as demonstrated by varied steroidogenic enzyme expression and elevated estradiol production. Thus, efforts should be made to avoid the use of implanted heifers to study steroidogenesis in small follicle GC culture systems.

## Competing interests

The authors declare they have no competing interest.

## Authors’ contribution

ADS carried out granulosa cell isolation and cell culture including treatment and collection of cells and media. ADS conducted real-time PCR gene expression analysis. DMH carried out the radioimmunoassays. CAG and JAHG were involved in experimental design, interpretation of data and writing of this manuscript. All authors read and approved the final manuscript.
